# Supplementary material for: IKKε Inhibitor Amlexanox Promotes Olaparib Sensitivity through the C/EBP-β-Mediated Transcription of Rad51 in Castrate-Resistant Prostate Cancer
Source: Cancers (Basel). 2022 Jul 28;14(15):3684. doi: 10.3390/cancers14153684 (PMC9367422; doi:10.3390/cancers14153684)
Supplement: Supplementary file 1 [file cancers-14-03684-s001.zip › cancers-1810584-supplementary.pdf]

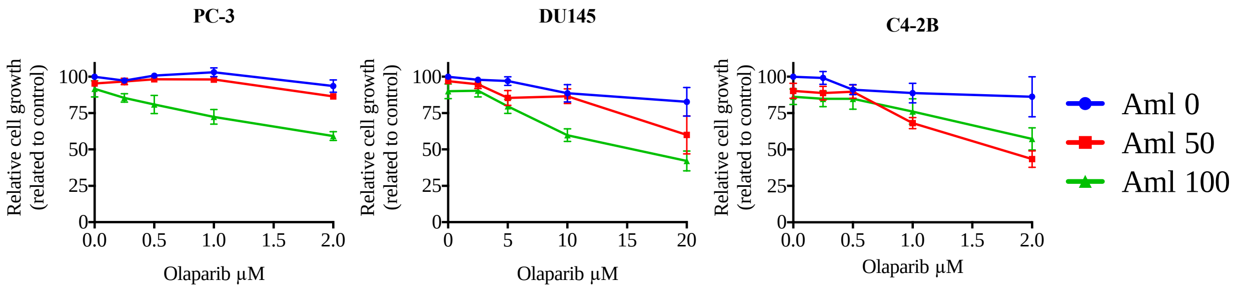

Figure S1. Optimizing inhibitory concentrations of Amlexanox-Olaparib combination in CRPC cell lines. Cell growth of CRPC cell lines under different concentrations of Olaparib combined with 50 μM or 100 μM Amlexanox (Aml) after 6 days.

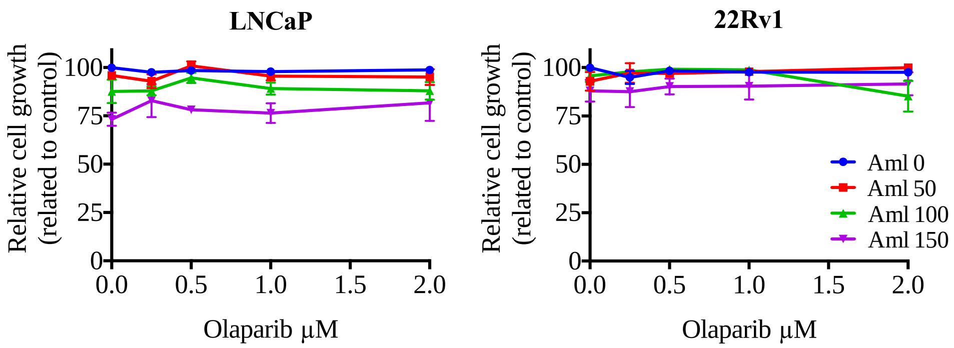

Figure S2. Amlexanox does not affect Olaparib sensitivity in HSPC cell lines. Cell growth of HSPC cell lines under different concentrations of Olaparib combined with 50, 100 and 150 μM Amlexanox (Aml) after 6 days.

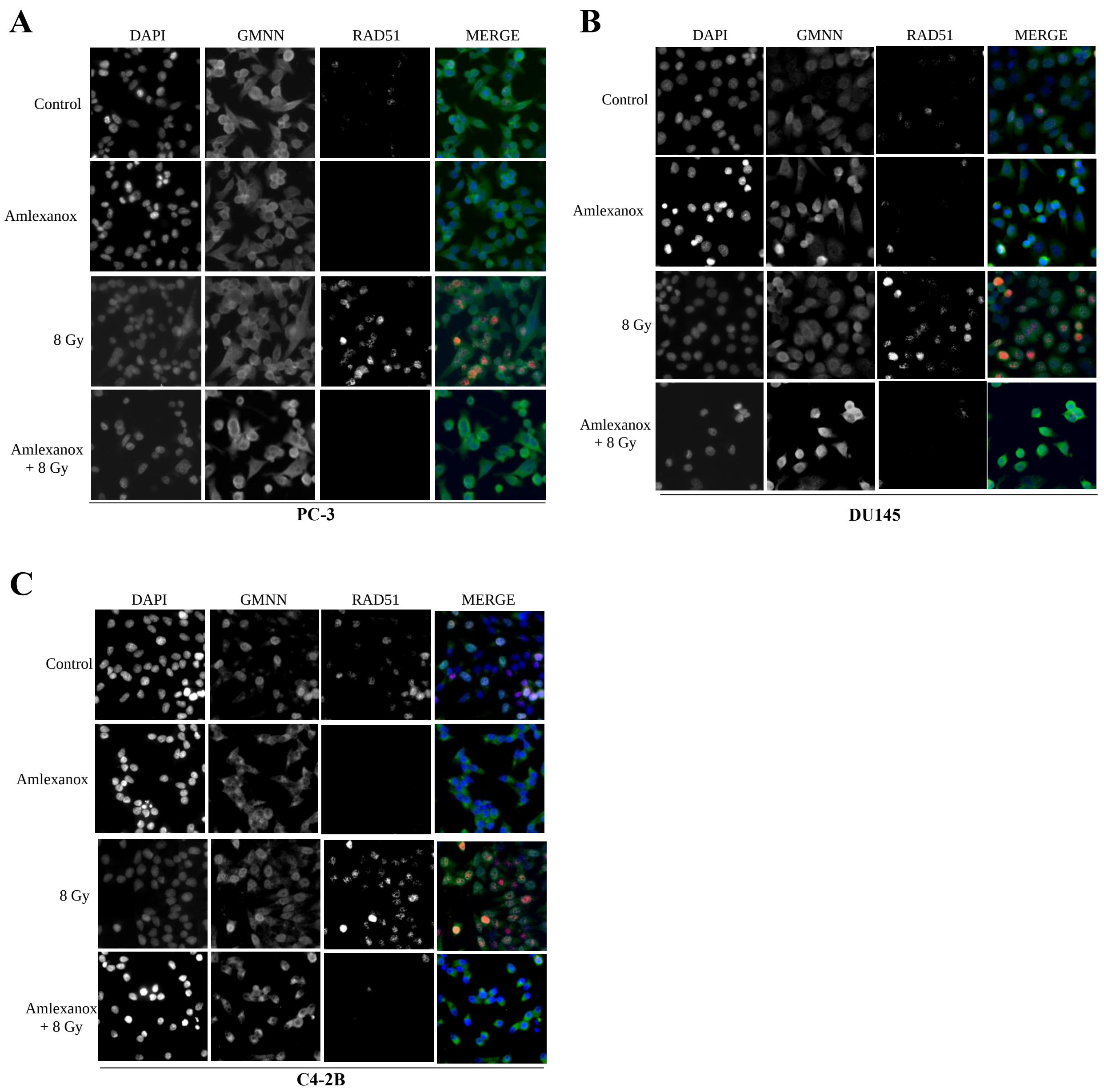

Figure S3. Amlexanox inhibits Rad51 recruitment induced by irradiation of CRPC cell lines. (A-C) Representative images of Rad51 foci recruitment after 2 hours of 8 Gy-irradiation in PC-3 (A), DU145 (B) and C4-2B (C) treated with or without 100 μM Amlexanox for 48 hours.

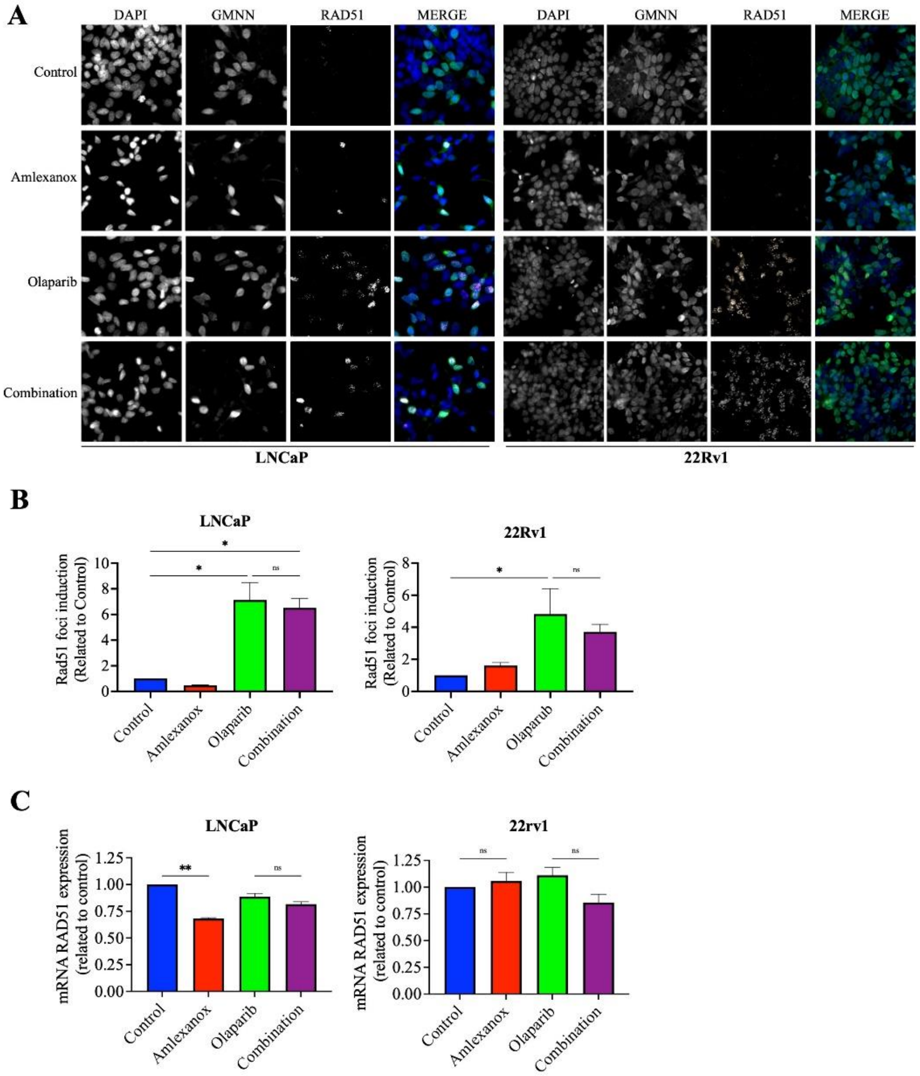

Figure S4. Amlexanox does not inhibit Olaparib-induced Rad51 recruitment and transcription in HSPC cell lines. (A-B) Representative images (A) and quantification (B) of Rad51 foci recruitment in HSPC cell lines treated with Amlexanox (for 48 hours) and/or Olaparib (for 24 hours). (C) Rad51 expression measured by RT-qPCR in HSPC cells treated with 100 μM Amlexanox and/or 10 μM Olaparib. Each experiment was repeated three times. Statistical significance was determined by two-way ANOVA. **p<0.01.

A

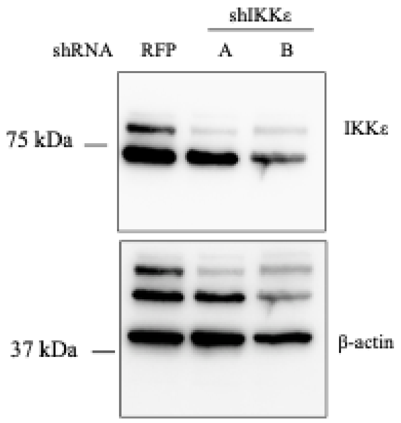
shIKKε
shRNA
RFP
A
B
75 kDa
IKKε
37 kDa
β-actin

B

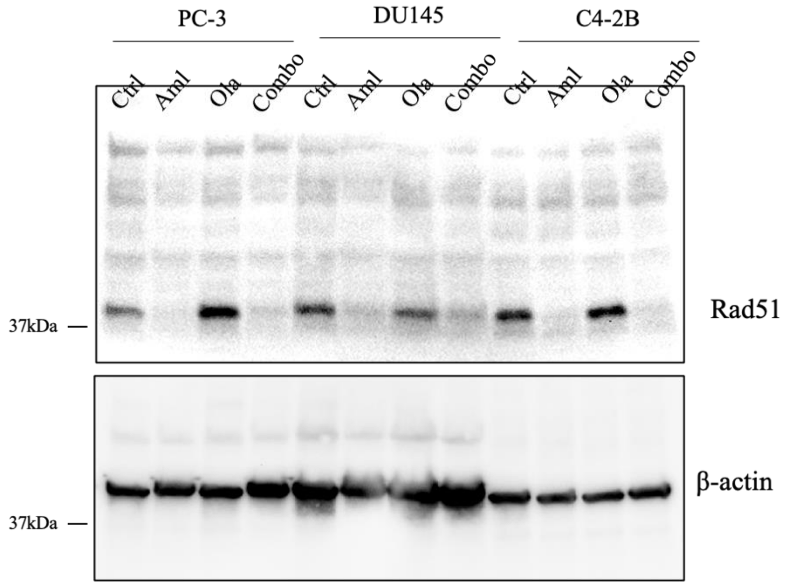
PC-3
DU145
C4-2B
Ctrl
Aml
Ola
Combo
Ctrl
Aml
Ola
Combo
Ctrl
Aml
Ola
Combo
37kDa
Rad51
37kDa
β-actin

C

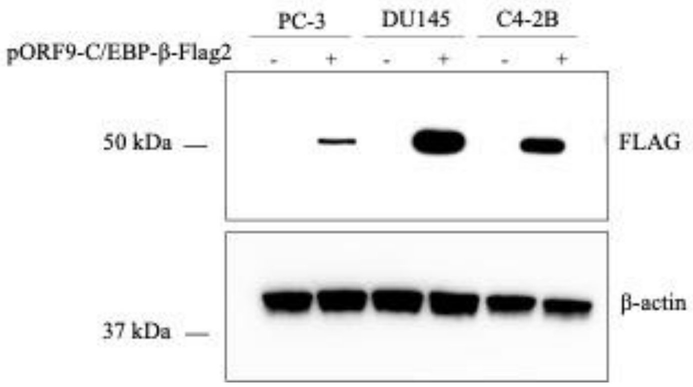
PC-3
DU145
C4-2B
pORF9-C/EBP-β-Flag2
- + - + - +
50 kDa
FLAG
37 kDa
β-actin

D

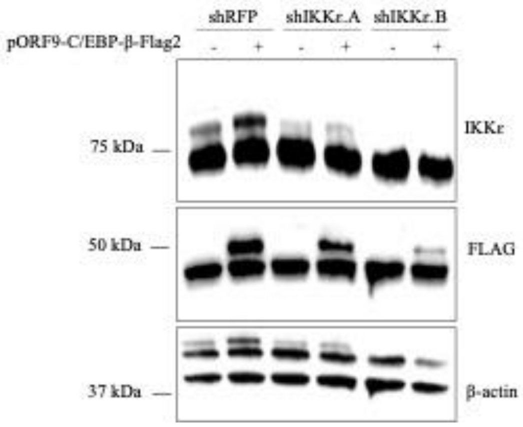

Figure S5. Original Western Blot images. (**A**) For Figure 3D. (**B**) For Figure 4B. (**C**) For Figure 5D. (**D**) For Figure 5F.
